# Supplementary material for: Supramolecular Atropine Potentiometric Sensor
Source: Sensors (Basel). 2021 Aug 31;21(17):5879. doi: 10.3390/s21175879 (PMC8434286; doi:10.3390/s21175879)
Supplement: Supplementary file 1 [file sensors-21-05879-s001.zip › sensors-1312168-supplementary.pdf]

sensors

Supplementary Material

Supramolecular Atropine Potentiometric Sensor

Catarina Ferreira [1], Andreia Palmeira [2,3], Emília Sousa [2,3], Célia G. Amorim [1,*], Alberto Nova Araújo [1] and Maria Conceição Montenegro [1]

1 LAQV/REQUIMTE, Departamento de Ciências Químicas, Faculdade de Farmácia, Universidade do Porto, R. Jorge Viterbo Ferreira 228, 4050-313 Porto, Portugal; up201305083@ff.up.pt (C.F.); anaraujo@ff.up.pt (A.N.A.); mcbranco@ff.up.pt (M.C.M.)
2 Laboratório de Química Orgânica e Farmacêutica, Faculdade de Farmácia, Universidade do Porto, Rua de Jorge Viterbo Ferreira 228, 4050-313 Porto, Portugal; apalmeira@ff.up.pt (A.P.); esousa@ff.up.pt (E.S.)
3 CIIMAR—Centro Interdisciplinar de Investigação Marinha e Ambiental, Terminal de Cruzeiros do Porto de Leixões, 4450-208 Matosinhos, Portugal
* Correspondence: camorim@ff.up.pt

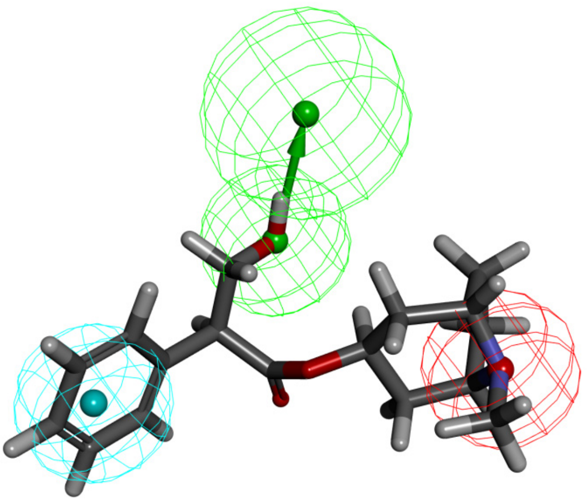

Figure S1. Common feature pharmacophore of test compound atropine (represented as sticks) and the six controls (ommited for simplification) obtained using the HipHop algorithm of Catalyst v16. The pharmacophore is composed of one aromatic ring (blue sphere), one hydrogen-bond donnor group (green spheres and arrow) and one positive ionizable group (red sphere).
